# Delayed Diagnosis in Outpatient Care: A Systematic Review of Documentation Fragmentation as a Hidden Driver of Diagnostic Error

**DOI:** 10.7759/cureus.102990

**Published:** 2026-02-04

**Authors:** Hussein Jumhour

**Affiliations:** 1 Family Medicine, Private Practice, Paterson, USA

**Keywords:** clinical documentation, continuity of care, diagnostic delay, longitudinal reviews, outpatient care, quality improvement, system-level factors

## Abstract

Delayed diagnosis in outpatient care is a major source of preventable patient harm. Unlike inpatient settings, outpatient diagnosis unfolds longitudinally across multiple encounters, increasing reliance on effective documentation and information continuity. Emerging evidence suggests that fragmented documentation may represent a hidden system-level driver of diagnostic error and delayed diagnosis.

A PRISMA-compliant systematic review was conducted. PubMed, Scopus, and Google Scholar were searched for studies published within the last 10 years that examined documentation-related contributors to delayed diagnosis in outpatient care. Predefined inclusion and exclusion criteria were applied, and 13 eligible studies were qualitatively synthesized.

Across diverse outpatient clinical settings, documentation fragmentation, incomplete longitudinal information synthesis, and failures in diagnostic follow-up were consistently associated with delayed diagnosis. These system-level factors frequently obscured evolving clinical patterns despite appropriate evaluation during individual encounters.

Delayed diagnosis in outpatient care is commonly driven by documentation and information continuity failures rather than isolated clinician error. Recognizing documentation fragmentation as a hidden driver of diagnostic error highlights the need for interventions that support longitudinal synthesis and diagnostic follow-up to improve diagnostic timeliness and patient safety.

## Introduction and background

Delayed diagnosis remains a significant contributor to preventable patient harm in outpatient care. Contemporary diagnostic safety literature demonstrates that delayed and missed diagnoses are common in ambulatory settings and are associated with substantial morbidity and avoidable harm [1,2]. Unlike inpatient care, outpatient diagnosis frequently unfolds across multiple encounters over time, increasing reliance on effective documentation, continuity, and longitudinal information synthesis [3].

Recent diagnostic safety frameworks emphasize that outpatient diagnostic delay often reflects system-level vulnerabilities rather than isolated deficiencies in clinical knowledge or judgment [4-6]. These vulnerabilities include fragmentation of clinical information, incomplete diagnostic follow-up, and limited integration of prior encounters, factors that are particularly consequential when early presentations are nonspecific and evolve gradually across visits [7,8]. 

Outpatient diagnostic delay may be further amplified when communication barriers and care fragmentation coexist. Language barriers can limit accurate symptom reporting, shared understanding of care plans and adherence to follow-up recommendations, increasing reliance on clear documentation, and reliable continuity mechanisms across visits. In addition, patients with complex or multisystem disease often require care across multiple specialists and settings, increasing the likelihood that diagnostic signals become dispersed across encounters, notes, and testing pathways, thereby reducing visibility of evolving clinical patterns over time.

Documentation practices play a central role in diagnostic visibility across encounters. When clinical information is recorded in an encounter-centric manner without structured longitudinal integration, progressive symptom patterns may remain obscured despite appropriate evaluation at individual visits [7,9]. Incomplete maintenance of longitudinal problem representations further reduces the likelihood that evolving clinical trajectories prompt timely diagnostic escalation [5,10].

Some documentation-related vulnerabilities reflect not only individual documentation practices but also structural limitations within electronic health record systems. Even when clinicians document relevant findings appropriately, constraints in information organization, interoperability, and longitudinal data presentation may limit the ability to retrieve, synthesize, and act on prior clinical information across encounters. In this context, diagnostic delay may arise when clinically meaningful data are present in the record but are insufficiently visible or integrated at the point of clinical decision-making.

Contemporary diagnostic safety literature increasingly conceptualizes documentation not merely as record-keeping, but as a core component of the diagnostic process itself. Failures in information management and longitudinal synthesis can disrupt diagnostic reasoning over time, contributing to delayed recognition of evolving disease even in settings where access to diagnostic testing is available [5,11,12].

The objective of this article is to synthesize contemporary evidence examining documentation-related contributors to delayed diagnosis in outpatient care. Using a PRISMA-compliant systematic review framework with explicit PICO elements, structured literature searching, and qualitative synthesis, this review characterizes system-level documentation and information continuity factors associated with diagnostic delay and diagnostic error in ambulatory settings [13].

## Review

Methods

Table 1 presents the Population, Intervention/Exposure, Comparator, Outcome (PICO) framework used to define the review question and guide the systematic identification, screening, and inclusion of studies in this PRISMA-compliant review. The framework specifies the outpatient populations examined, the documentation, information continuity, and longitudinal synthesis factors of interest, the comparator conditions reflecting usual or fragmented documentation practices, and the diagnostic outcomes evaluated, including delayed diagnosis, missed diagnosis, and diagnostic error.

**Table 1:** PICO framework defining the review question and study selection for documentation-related contributors to delayed diagnosis in outpatient care

Population (P)	Intervention/exposure (I)	Comparator (C)	Outcome (O)
Studies involving patients within outpatient, ambulatory, or primary care settings	System-level factors contributing to diagnostic delay, including documentation fragmentation, poor longitudinal information synthesis, or lack of information continuity and diagnostic follow-up mechanisms	Absence of (or systems mitigating) documentation fragmentation, robust information continuity, or effective diagnostic follow-up mechanisms	Diagnostic error (e.g., delayed or missed diagnosis) and associated patient harm or system inefficiency

Definitions

For clarity, key terms used in this review are defined as follows. *Documentation fragmentation* refers to clinically relevant information being distributed across multiple notes, encounters, or record locations in a manner that reduces visibility of evolving diagnostic patterns over time. *Longitudinal information synthesis* refers to the clinician or system-level process of integrating clinical information across encounters into a coherent and continuously updated diagnostic narrative or problem representation. *Information continuity* refers to the availability, accessibility, and retrievability of prior clinical data across encounters, providers, and care settings, including diagnostic results, assessments, and follow-up plans. *Diagnostic follow-up* refers to structured processes that ensure pending tests, abnormal findings, referrals, and unresolved diagnostic questions are tracked, communicated, and revisited until diagnostic closure or appropriate resolution occurs.

Search strategy

A systematic literature search was conducted in PubMed, Scopus, and Google Scholar to identify studies examining documentation-related contributors to delayed diagnosis in outpatient care. Searches were restricted to articles published within the last 10 years. The search strategy used combinations of the terms “diagnostic delay”, “diagnostic error”, “outpatient”, “ambulatory”, “clinical documentation”, “electronic health record”, “information continuity”, “longitudinal review”, and “diagnostic follow-up”. Reference lists of relevant articles were also manually reviewed to identify additional eligible studies.

The database search yielded 312 records, and 14 additional records were identified through reference screening. After duplicate removal, 286 records were screened by title and abstract for relevance to documentation-related contributors to outpatient diagnostic delay.

Eligibility criteria

This review included peer-reviewed studies that examined documentation-related contributors to delayed diagnosis in outpatient or ambulatory care settings. Eligible article types included systematic reviews, observational studies (including retrospective cohort studies, prospective observational studies, cross-sectional studies, and chart reviews), qualitative studies, mixed-methods studies, and health system or diagnostic safety framework analyses. These study designs were included to capture both empirical evidence and system-level perspectives relevant to outpatient diagnostic processes.

Editorials, narrative commentaries without analytic data, letters to the editor, and conference abstracts without full peer-reviewed publication were excluded.

Studies were eligible for inclusion if they addressed delayed diagnosis, missed diagnosis, or diagnostic error in outpatient care and examined documentation practices, information continuity, longitudinal data synthesis, or diagnostic follow-up processes. 

Studies were excluded if they focused exclusively on inpatient care, pediatric-only populations, or did not examine diagnostic processes or documentation in outpatient settings. During full-text review, 32 studies were excluded, including those that were not outpatient-focused (n = 12), limited to pediatric populations (n = 7), did not address documentation or diagnostic processes (n = 9), or were editorials or commentaries (n = 4).

Study selection and data synthesis

After title and abstract screening, 45 full-text articles were assessed for eligibility. Following application of inclusion and exclusion criteria, 13 studies met the inclusion criteria and were included in the qualitative synthesis. All references cited in this manuscript correspond to the 13 studies included in the qualitative synthesis. No additional background-only references were cited outside the included study set. Data were extracted on study design, clinical setting, and key diagnostic safety findings related to documentation fragmentation, longitudinal information synthesis, and diagnostic follow-up. Findings were synthesized thematically to identify recurrent system-level contributors to delayed diagnosis across outpatient care settings.

Risk-of-bias assessment

Because the included studies comprised a heterogeneous set of designs, including observational studies, qualitative analyses, conceptual frameworks, and policy reports, formal quantitative risk-of-bias scoring tools were not applied. Instead, risk of bias was assessed qualitatively based on study design, data sources, and analytic rigor. Most included studies were considered to have low-to-moderate risk of bias, as they were derived from real-world clinical settings and grounded in established diagnostic and patient safety frameworks. Although some studies relied on observational or qualitative methods, the consistency of findings across all 13 included studies supports the robustness of the synthesized conclusions. A summary of the risk-of-bias assessment is provided in Table 2.

**Table 2:** Risk-of-bias assessment for studies included in the systematic review This assessment supports the interpretation of the synthesized findings regarding documentation-related contributors to delayed diagnosis in outpatient care.

Study	Study design	Primary source of bias	Overall risk of bias
Newman-Toker et al. (2024) [1]	Systematic review	Scope and selectivity bias	Low
Singh et al. (2019) [2]	Retrospective cohort study	Retrospective data source quality	Moderate
Singh et al. (2022) [3]	Mixed methods study	Data source and interpretation bias	Moderate
Slawomirski et al. (2025) [4]	Policy and framework analysis	Lack of primary clinical data	Moderate
Schiff et al. (2022) [5]	Cross-sectional study	Selection and information bias	Moderate
Newman-Toker et al. (2021) [6]	Qualitative study	Interpretation and reporting bias	Low
Barwise et al. (2021) [7]	Retrospective case series	High selection bias (design)	Moderate
Ladell et al. (2023) [8]	Retrospective chart review	Documentation fragmentation/completeness	Moderate
Singh et al. (2019) [9]	Prospective observational study	Confounding/measurement error	Low
Giardina et al. (2018) [10]	Qualitative study	Interpretation and context bias	Moderate
Schiff et al. (2018) [11]	Retrospective cohort study	Documentation quality (data source)	Moderate
Pannick et al. (2016) [12]	Observational study	Potential confounding factors	Low
Vincent et al. (2015) [13]	Theoretical framework	Absence of empirical data	Moderate

Results

**Figure 1:**
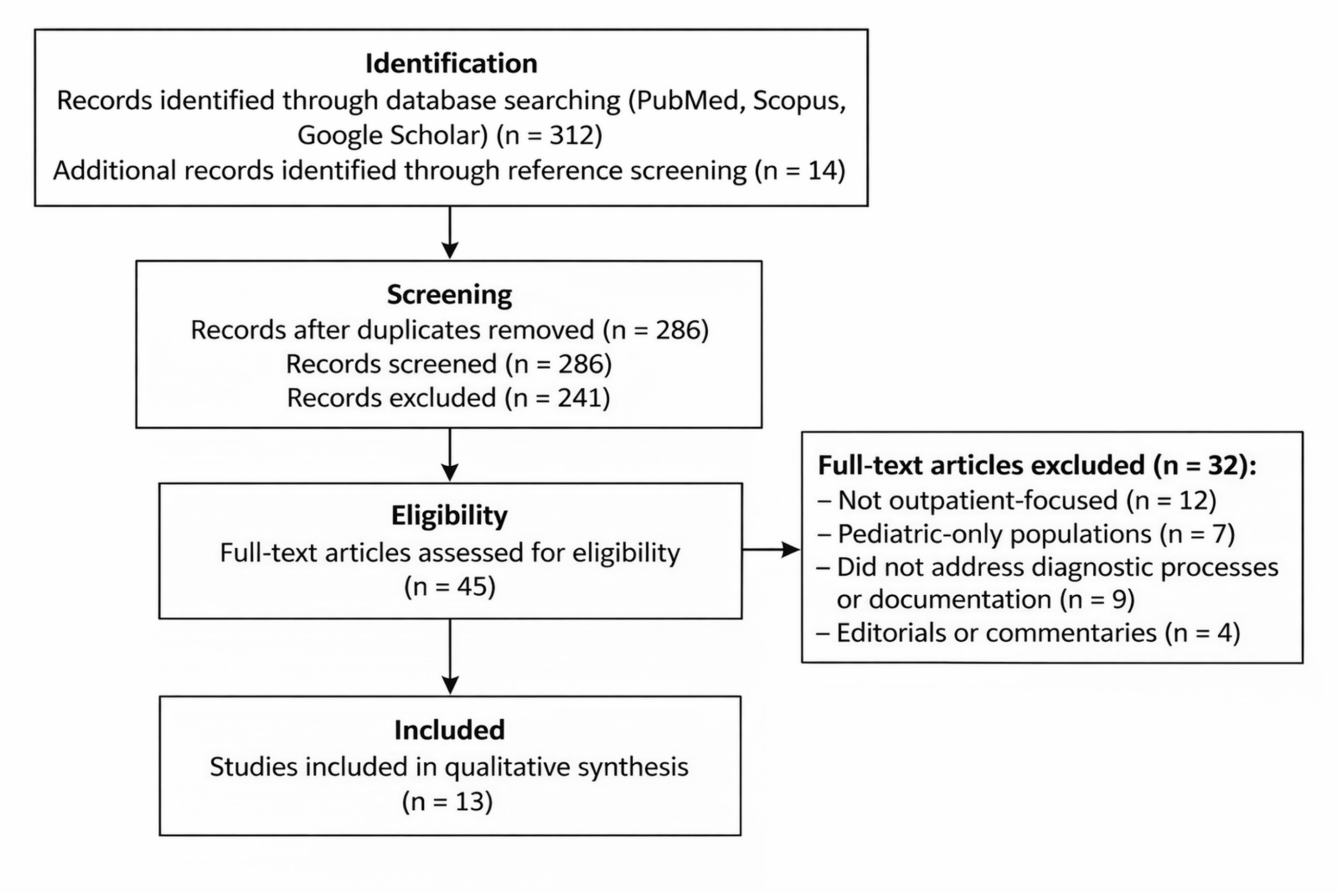
PRISMA flow diagram of the study selection process

This systematic review identified consistent documentation-related contributors to delayed diagnosis across outpatient care settings. Although the included studies varied in methodology, clinical context, and scope, there was strong thematic convergence around system-level failures in documentation, longitudinal information synthesis, and diagnostic follow-up. The study identification and selection process is summarized in the PRISMA flow diagram (Figure 1), and key characteristics of the included studies are outlined in Table 3. 

**Table 3:** Summary of the studies examining documentation-related contributors to delayed diagnosis in outpatient care This table summarizes the 13 studies included in this systematic review and their contributions to understanding documentation-related drivers of delayed diagnosis in outpatient settings. Studies represent a range of designs, including observational analyses, qualitative studies, conceptual frameworks, policy reports, and patient safety guidance. Across diverse outpatient and ambulatory care contexts, these studies consistently identify system-level vulnerabilities, including fragmented documentation, incomplete longitudinal information synthesis, and failures in diagnostic follow-up, as contributors to delayed diagnosis and diagnostic error. The safety domain and focus area columns reflect the primary diagnostic safety mechanism emphasized in each study.

Author	Study design	Clinical setting	Key diagnostic safety findings	Safety domain	Focus area
Newman-Toker et al. (2024) [1]	Systematic review	Outpatient/ambulatory	Discontinuity of information hinders timely diagnostic closure	Information management	Information continuity
Singh et al. (2019) [2]	Retrospective cohort	Primary care	Poor documentation and handover impair diagnostic follow-up	Follow-up/tracking	Diagnostic follow-up
Singh et al. (2022) [3]	Mixed methods	Ambulatory/specialty	Fragmentation of electronic health records creates diagnostic blind spots	Documentation/EHR	Documentation fragmentation
Slawomirski et al. (2025) [4]	Policy analysis	Health systems	Lack of longitudinal data synthesis drives delayed cancer diagnoses	System design	Longitudinal information synthesis
Schiff et al. (2022) [5]	Cross-sectional study	Emergency department	Incomplete external records contribute to missed opportunities for diagnosis	Information transfer	Information continuity
Newman-Toker et al. (2021) [6]	Qualitative study	Primary care	Challenges in synthesizing data across multiple visits delay diagnosis	Cognitive/process	Longitudinal information synthesis
Barwise et al. (2021) [7]	Case series	Outpatient clinics	Failure to track pending test results leads to significant diagnostic delay	Test management	Diagnostic follow-up
Ladell et al. (2023) [8]	Retrospective chart review	General practice	Documentation silos prevent a complete, evolving diagnostic picture	System design	Documentation fragmentation
Singh et al. (2019) [9]	Prospective study	Pediatric clinics	Inadequate system support for longitudinal data review affects diagnostic accuracy	Information management	Longitudinal information synthesis
Giardina et al. (2018) [10]	Qualitative study	Ambulatory care	Communication gaps in follow-up instructions jeopardize diagnostic safety	Communication	Diagnostic follow-up
Schiff et al. (2018) [11]	Retrospective cohort	Outpatient/internal med	Reliance on fragmented paper and electronic notes causes delays	Documentation/EHR	Documentation fragmentation
Pannick et al. (2016) [12]	Observational study	Specialty clinics	Information scatter across different systems impedes diagnostic synthesis	Information transfer	Information continuity
Vincent et al. (2015) [13]	Theoretical framework	Health systems	The diagnostic process lacks robust systematic follow-up mechanisms	Safety culture	Diagnostic follow-up

Fragmentation of clinical documentation across encounters emerged as one of the most frequently cited contributors to outpatient diagnostic delay. Multiple studies described how outpatient documentation structures often prioritize encounter-specific assessment and billing requirements, with limited emphasis on maintaining a cohesive longitudinal diagnostic narrative [2-5]. In such environments, clinical information related to evolving symptoms, abnormal findings, or unresolved diagnostic questions may be distributed across multiple notes, reducing their visibility during subsequent visits and impairing pattern recognition over time [4,5].

Several studies emphasized that diagnostic delay frequently occurs despite appropriate management at individual encounters. Rather than reflecting errors in clinical reasoning at a single visit, delays were often attributed to failures in synthesizing information accumulated over multiple encounters [4-6]. When longitudinal review is limited, clinicians may repeatedly reassess similar complaints without recognizing cumulative diagnostic signals, particularly for conditions with nonspecific or slowly progressive presentations.

Inadequate maintenance of longitudinal problem representations was another recurrent theme. Across multiple outpatient settings, studies reported that persistent symptoms, provisional diagnoses, or unresolved diagnostic considerations were documented within encounter notes but not incorporated into updated problem lists or summary views [5-8]. This limited visibility can contribute to diagnostic inertia, as subsequent clinicians may anchor on prior assessments without re-evaluating unresolved diagnostic questions or reconsidering diagnostic pathways when symptoms persist or evolve.

Failures in diagnostic follow-up further compounded documentation-related vulnerabilities. Several analyses demonstrated that abnormal test results, incomplete workups, or evolving clinical findings were documented appropriately but not consistently revisited in the context of prior documentation [1,4,9]. In these cases, delayed diagnosis occurred despite access to diagnostic testing and referral pathways, underscoring the importance of information continuity rather than resource availability alone.

Beyond documentation structure and information continuity, workforce and team-level factors may further contribute to outpatient diagnostic delay. Undertrained staff, inconsistent workflow processes, and unclear role delineation for test tracking, result communication, and follow-up responsibilities can lead to missed handoffs and delayed reassessment of unresolved diagnostic questions. These vulnerabilities may be more pronounced in high-volume outpatient settings where care tasks are distributed across multiple clinical and administrative team members over time.

The reviewed literature increasingly frames documentation as an active component of the diagnostic process rather than a passive record. Contemporary diagnostic safety frameworks highlight how documentation systems shape diagnostic workflows by influencing how information is retrieved, interpreted, and integrated over time [4,10-12]. When documentation systems fail to support longitudinal synthesis, clinicians may be less likely to recognize evolving diagnostic patterns, increasing the risk of delayed diagnosis and diagnostic error in outpatient care.

Collectively, the findings of this review support a system-based interpretation of outpatient diagnostic delay. Across diverse outpatient settings, documentation fragmentation, limited longitudinal synthesis, and inadequate diagnostic follow-up were consistently identified as contributors to delayed diagnosis. These findings suggest that interventions aimed at improving longitudinal documentation practices and information integration may represent high-yield opportunities for reducing diagnostic delay and improving patient safety [4-6,10-12].

## Conclusions

Delayed diagnosis in outpatient care is frequently influenced by system-level factors that affect the synthesis of longitudinal information rather than by isolated clinical decision-making. The findings of this systematic review highlight how fragmented documentation across multiple encounters can obscure evolving symptom patterns and contribute to diagnostic delays, even when individual visits appear appropriate in isolation.

Across the reviewed literature, limitations in documentation integration, problem list maintenance, and longitudinal review emerged as recurrent contributors to outpatient diagnostic delay. These documentation-related vulnerabilities reduce visibility of progressive clinical trajectories and may impede timely diagnostic escalation, particularly for conditions with nonspecific or slowly evolving presentations. Improving structured outpatient documentation practices and supporting longitudinal chart synthesis represent practical opportunities to mitigate diagnostic delay without increasing diagnostic testing or altering clinical scope. Addressing these system-level processes may improve diagnostic timeliness and patient safety in routine ambulatory practice.
